# The role of the intraparietal sulcus in numeracy: A review of parietal lesion cases

**DOI:** 10.1016/j.bbr.2025.115453

**Published:** 2025-01-30

**Authors:** Erin Duricy, Corrine Durisko, Julie A. Fiez

**Affiliations:** a Learning Research and Development Center, University of Pittsburgh, Pittsburgh, PA 15260, USA; b Center for Neuroscience, University of Pittsburgh, Pittsburgh, PA 15260, USA; c Center for the Neural Basis of Cognition, University of Pittsburgh, Pittsburgh, PA 15260, USA; d Department of Psychology, and, University of Pittsburgh, Pittsburgh, PA 15260, USA; e Department of Communication Science and Disorders, University of Pittsburgh, Pittsburgh, PA 15260, USA

**Keywords:** Acalculia, Stroke, Numeracy, Neuropsychology, Intraparietal sulcus, Parietal

## Abstract

Prominent theories of numeracy link the intraparietal sulcus (IPS) to approximate representations of quantity that undergird basic math abilities. The goal of this review is to better understand the neural basis of mathematical cognition through the lens of acalculia, by identifying numeracy-focused single case studies of patients with parietal lesions and testing for causal relationships between numeracy impairments and the locus of parietal damage. A systematic literature review identified 27 single case studies with left parietal lesions and categorized administered tasks across four numeracy domains: Approximation, Calculation, Ordinality/Cardinality, and Transcoding. We compared published lesion images by drawing a sphere at the inferred center-of-mass and assigning each case to an anatomical group (IPS or Other Parietal damage) based on overlap with left IPS and original anatomical description. We performed Fisher’s Exact Test to compare behavioral performance on each numeracy domain between the two groups. As an exploratory follow-up, we used Activation Likelihood Estimation (ALE) to identify sites of damage within parietal cortex preferentially associated with impairments in each domain. We found that Approximation impairments were significantly more frequent in the IPS group (p = .003). The exploratory ALE analysis revealed that only Approximation impairment cases significantly overlapped with the IPS, while impairments in other domains were localized to different regions of the parietal lobe. Based on the pattern of impairments shown across these cases, we conclude that damage to the left IPS is linked to impairments in approximation ability specifically. Our findings support theoretical claims linking IPS to magnitude representation, but do not provide evidence that IPS critically underpins performance across all numeracy tasks. Instead, our findings are more compatible with models of dissociable circuits of numerical processing within the parietal lobe.

## 1. Introduction

Numeracy, the ability to understand and manipulate numbers, is a crucial skill required for daily life. The ability to assess magnitude and employ numerical operations allows us to read a clock or thermometer, complete a monetary transaction, or understand quantities in tasks such as cooking. Because the use of numbers is such a large part of modern society, impairment in numeracy abilities can lead to a decrease in overall quality of life through employment, social, and emotional effects [22,76,92]. In fact, many stroke survivors with acquired numeracy deficits, or acalculia, report emotional distress, shame, and a lack of daily life independence as a result of acalculia [13]. Therefore, numeracy and its neurological underpinnings have been explored for decades to better understand how numerical cognition is achieved and how numerical deficits arise.

The Triple Code Model posited by Dehaene and colleagues (1992,1995) remains the most prevalent theory to explain numerical processing in the brain and has largely shaped the direction of numeracy literature in both behavioral and neuroanatomical domains. It has served as a foundation for the prevailing theory that the bilateral intraparietal sulcus (IPS) is a key region in numeracy due to its central role in magnitude processing [20,40,86,96]. Additional support for the IPS as a core of magnitude processing arises from the Approximate Number System theory, in which the IPS is proposed to underlie an innate understanding of magnitude upon which learned math skills are formed [36,91]. These theories have been investigated through both functional methodologies and loss-of-function approaches, in which behavioral impairments are investigated following damage to a specific brain region. The loss-of-function studies have often taken the form of a single case study. Single case studies report on one individual case (or a small collection of cases) of disordered processing ability with a high degree of detail that can help reveal underlying mechanisms and test theoretical predictions. Many case studies have been published describing patterns of numeracy performance following focal brain injury, but to date these single cases have not been compiled to examine the similarities and differences between them, or how their neuroanatomical findings compare to the prevailing theories of numerical processing. This review aims to fill that gap through an investigation of published single case studies, with a focus on the IPS and its role as described in the Triple Code Model and Approximate Number System theoretical perspectives.

### 1.1. Theoretical background

Dehaene first proposed the Triple Code Model in 1992 as an alternative to McCloskey’s model of numerical cognition [35,87]. The Triple Code Model describes three primary representations of numeracy: *visual Arabic number form, verbal auditory word frame,* and *analog magnitude representation* [35,38]. The *visual Arabic number form* code, localized to the bilateral inferior ventral occipitotemporal region, is a representation of numerical symbols in a visuo-spatial manner so that they may be input through reading and output in the form of Arabic numeral writing [38,39]. This representational code is thought to underlie reading and writing of digits, as well as the ability to perform multi-digit operations and parity decision-making. The *verbal auditory word frame* code*,* localized to left hemisphere perisylvian area, is a representation of the word form of number. It allows for abilities such as counting and arithmetic fact retrieval (e.g., multiplication tables), in the form of bidirectional written and oral production. The *analog magnitude representation* code, localized bilaterally in the inferior parietal lobe (particularly in the IPS), is a pre-verbal representation that encompasses approximate reasoning through magnitude comparison and approximate calculation [38,39]. It allows for abilities such as estimation and subitizing (the ability to perceive small quantities without counting) [72].

Analog magnitude representations supported by the IPS have been central to further theories of an innate Approximate Number System found across species [52,57,88,91,123]. The Approximate Number System is thought to form a “number sense” present at birth that gives rise to basic understanding of magnitude, distinct from verbal or visual symbolic representation [36]. In fact, Approximate Number System acuity has been linked to stronger arithmetic performance in children as early as preschool [125]. Thus, in humans, innate magnitude approximation ability is thought to underlie learned symbolic mathematical concepts, such as calculation, transcoding, and cardinality, and is considered central to all forms of numerical processing [57,91].

Importantly, the Triple Code Model and Approximate Number System theories concur on the concept of a foundational approximate number representation. Both perspectives suggest that the IPS is involved in nonverbal magnitude processing and allows for the approximation and comparison of quantities. In turn, this skill underlies performance on other numerical tasks. In the Triple Code Model, the IPS is described as a core for the semantics of number and therefore crucially supports symbolic, exact number abilities like calculation. In the Approximate Number System, the approximate magnitude representation role of the IPS serves developmentally as a foundation for symbolic number processing learning to occur. Overall, the ability to approximate magnitude is considered to be central to mathematical processing as a whole.

Interestingly, after the publication of their Triple Code Model [35], Dehaene and colleagues (2003) later proposed three numeracy regions with separable functionality all located within the parietal lobe as an expansion of the initial hypothesis. This modification of the Triple Code Model elaborates additional regions of the parietal lobe that contribute to numerical processing, in coordination with the representational coding originally proposed. In this later publication, the bilateral IPS remained the key region for understanding of nonverbal quantity. The left angular gyrus was identified as a region associated with verbal processing of numeracy, particularly math facts achieved through rote verbal memory, while the bilateral posterior superior parietal cortex was suggested as a visuospatial element involved in spatial attention-orienting upon the number line [41]. This modification has been less explored than the original Triple Code Model hypothesis implicating only the IPS in the semantic number code, but the proposed parietal areas have been highlighted in separate functional studies [49,104,108,116,131]. The left angular gyrus has been particularly associated with arithmetic fact retrieval and solving basic calculations [61,62, 102,112,131]. Similarly, the posterior parietal cortex has been implicated across calculation subtypes (e.g., addition, subtraction) [104, 108]. Such evidence supports the possibility that numerical processing integrates multiple regions of the parietal cortex.

### 1.2. Hemispheric differences across IPS and parietal cortex

The vast majority of IPS research studies since the development of the Triple Code Model have employed functional magnetic resonance imaging (fMRI) techniques to investigate IPS activation during specific numeracy tasks and with different types of stimulus formats. The results strongly suggest bilateral involvement of the IPS across facets of numerical processing, along with evidence of hemispheric specialization that emerges over time. Consistently, the left IPS has been strongly implicated in symbolic number processing across modalities and throughout development [20,86,129], though there is also evidence supporting the involvement of the right IPS in symbolic number processing [83,103,128]. Further, both left and right IPS are linked to non-symbolic magnitude processing through dot estimation and comparison tasks, often with patterns of greater activation in the right IPS specifically [3,16,68,69,83,128]. For example, Venkatraman et al. [128] investigated IPS fMRI activity during symbolic and non-symbolic addition tasks, and found that the bilateral anterior IPS and left posterior IPS showed the same activation for both stimulus formats. Similarly, Lyons et al. [83] investigated IPS activation during both symbolic and non-symbolic number comparison and found that the bilateral IPS demonstrated activity regardless of stimuli presentation, but that the specific coding of each format differed through independent tuning curves. A recent meta-analysis analyzed studies of numerical cognition that utilized symbolic and non-symbolic tasks and found bilateral IPS activation across both formats, with a degree of format-dependent lateralization [48]. Further, the open-access meta-analytic platform Neurosynth [132] compiles results from fMRI studies for use, and a search of numeracy terms (e.g., magnitude, calculation) demonstrates convergent evidence for the reliable bilateral engagement of the IPS in various numeracy tasks. Developmentally, the IPS has been suggested as a foundational region of non-symbolic processing, with consistent activation of the right IPS through childhood to adulthood and an increase in left IPS activation into adulthood as specialization may occur [23].

As a complement to the findings of functional imaging in neurologically-intact samples, several neuropsychological case series have compared groups of left hemisphere (LH) versus right hemisphere (RH) lesion cases [44,63,79,109]. Grafman et al. [63] assessed 41 LH and 35 RH lesion participants on written calculation performance, finding that LH participants performed significantly more poorly than RH participants, and those with LH posterior damage showed greater overall impairment than the individuals with LH anterior damage. Rosselli & Ardila [109] assessed acalculia in a sample of 41 LH and 21 RH lesion participants, with tasks including transcoding, written and mental arithmetic, counting, word problems, and aligning numbers in columns based on place value. They found significantly more frequent impairments in LH damage participants for tasks involving reading numbers and performing multiple operations, but not in magnitude comparison. Across tasks, LH retro-Rolandic participants demonstrated the most significant number of errors, relative to LH pre-Rolandic cases. RH cases more often demonstrated spatial errors or neglect in number column alignment and procedural carrying abilities, and did not demonstrate a significant difference between pre- and retro-Rolandic cases [109]. Langdon & Warrington [79] investigated hemispheric differences in arithmetic with a sample of 39 LH and 38 RH lesion participants assessed through the Graded Difficulty Arithmetic (GDA) and Arithmetical Reasoning Tests (ART). They found that LH damage participants performed significantly worse on the GDA, a measure of addition and subtraction, but that both LH and RH damage groups performed equally poorly on the ART, which required completing a series of numbers based on patterns of magnitude (e.g., 2, 4, 8, _) [79]. Dellatolas et al. [44] compared the performance of 56 LH and 24 RH brain-damaged participants on the EC301 numeracy battery. In this sample, LH lesion participants had greater impairments in verbal and written counting, transcoding, and calculation tasks, while RH lesion participants showed a greatest deficit in estimation and number line tasks [44].

Taken together, LH damage cases consistently demonstrated greater impairment on tasks involving calculation, transcoding, and reading or writing modalities, while RH damage cases more often demonstrated impairments of spatial skills and abstract magnitude reasoning. Studies with further neuroanatomical classifications, segmenting the brain into anterior/pre-Rolandic or posterior/retro-Rolandic regions, demonstrate the importance of the posterior region of the brain, in particular, to numeracy abilities. These results are consistent with functional evidence of the bilateral parietal cortex’s role in numerical processing, yet are limited due to the broad nature of the segmentations. To assess acalculia with a higher degree of neuroanatomical precision, one may turn to the single case study.

### 1.3. Case studies in numeracy

Since early studies of human cognition, one method for investigating the relationship of specific brain regions and behavioral phenotypes is to conduct a case study of an individual (or small group of individuals) with focal brain injury [1,50]. Using neuropsychological tests to probe cognitive performance in reference to a known brain abnormality, researchers can make inferences about the causality of the behavioral deficit relative to the specific site of brain damage. In contrast to case series, single-case studies most often provide a written anatomical description of the lesion location, along with one or more neuroanatomical figures depicting the site of lesioned tissue. In the numeracy literature, the identification of individuals with numeracy impairments following brain injury is complicated by the lack of clinical assessment for acalculia [13]. It is common for clinicians to employ neuropsychological assessments of aphasia to determine language or communication difficulties post-stroke, but these assessments do not typically probe math ability beyond optional brief subtests [7]. The EC301 is the most commonly cited standardized assessment of numerical performance [34, 44,45], and development of new clinical assessments of math has increased in the past two decades [5,43,100], but the vast majority of single cases studies in the literature do not utilize these tools. Therefore, reported cases of acalculia have often been detected as an impairment in calculation performance on limited subtests and, once acalculia has been identified, a non-standardized mix of assessments are conducted for additional components of numeracy beyond calculation. This includes assessments of approximation (magnitude estimation), transcoding (information conversion between modalities), and ordinality/cardinality (counting and number sequences). Such testing has resulted in a rich collection of behavioral data from a broad, though variable, range of numeracy tasks across single case studies.

Insightful information can be derived from the body of published single case studies when they are considered as a whole [50,111]. Outside of numeracy research, several recently published reviews have compiled lesioned single case studies in their respective fields to successfully analyze the cases as a sample, demonstrating the benefit of compiling preexisting cases for a broader goal [17,55,71,75]. However, the existing pool of single case study data in numerical processing remains underutilized. Individual reports highlight interesting behavioral patterns of acalculia, but are often isolated in the literature.

### 1.4. Summary of review

This review aims to fill a gap in numeracy literature by compiling numeracy-focused single case studies for direct comparison and application to predominant theories of numerical processing. Based on the prevailing view that the parietal cortex, and in particular the IPS, is central to magnitude processing, this review focuses on lesion cases involving parietal lobe damage to further elucidate the impact of IPS/parietal loss-of-function on numeracy ability. To achieve this focus, we report the results a systematic search for case studies investigating numeracy performance following a focal parietal lesion. In the compiled set of cases, we compare the frequency of numeracy impairments between parietal cases with or without IPS specific damage (for simplicity, termed IPS or Other Parietal cases). Based on prior imaging and neuropsychological literature, we expected to find a relatively even distribution of left and right hemisphere lesion cases with acalculia, with hemispheric differences as a function of the type of numerical task. On the basis of the Triple Code Model and Approximate Number System theories, lesions to the bilateral IPS should demonstrate a greater frequency of impairment in approximation ability. Because magnitude representation is thought to underlie other numeracy domains, IPS cases should also demonstrate greater frequency of impairment in tasks involving other components of numeracy.

## 2. Materials and methods

**Fig. 1.**
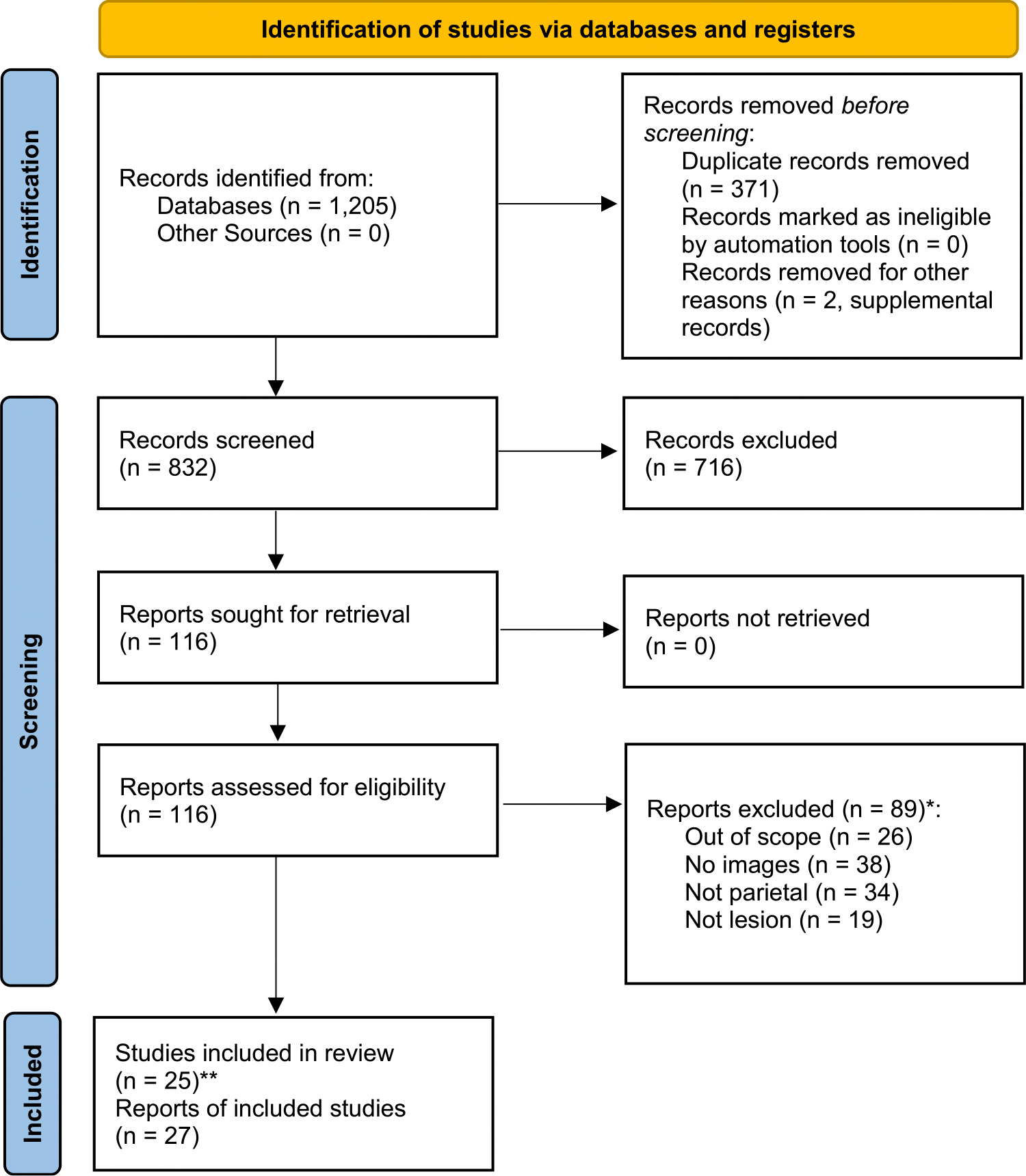
PRISMA Flow Chart for identification of studies for meta-analysis. *Many reports contained more than one reason for exclusion, and these counts are reflected below. **One study [118] contained three single cases that were considered individually. One case, patient JD, was assessed on three separate occasions [32,54,58], so two additional reports exist on this study.

In this review, case studies were systematically acquired by searching for cases addressing mathematical concepts and involving focal lesions to the parietal lobe caused by a cerebrovascular accident across the following databases: PubMed, Web of Science, and PsychInfo. In December 2022, an initial search included the following terms: (intraparietal OR intraparietal sulcus OR parietal) AND (math OR mathematics OR arithmetic OR calculation OR “number processing” OR approximation OR magnitude OR addition OR multiplication OR number) AND (case OR patient OR lesion). A second search in January 2023 used the same terms but replaced the specification of brain region with: (acalculia OR dyscalculia OR Gerstmann) in order to encompass articles without a focus on lesion location. The results from all database searches were imported to PICO Portal, a literature review synthesis platform [97]. All articles were screened and analyzed first at the abstract level to remove any results that were not case studies or numeracy-related, then analyzed at the full text level by author E.D. to confirm adherence to the inclusion criteria. All abstracts that exhibited uncertainty in meeting the defined inclusion criteria were discussed with authors C.D. and J.F., and a group consensus was reached prior to inclusion or exclusion. Fig. 1 depicts the identification of cases through the Preferred Reporting Items for Systematic Reviews and Meta-Analyses (PRISMA) flow chart [93].

### 2.1. Case selection

To be included in the current review, a source was required to meet the following inclusion criteria: 1) be a single case study (or collection of single cases) with a focal lesion in or near the parietal lobe, 2) involve a numeracy-focused assessment of math skills, and 3) contain at least one brain image to indicate the lesion location beyond written description. Based on this criteria, 27 single cases were included for further analysis ([10,11,15,25,27,30–32,37,42,54,58,66,67,78,80,81,85,94,99]; E.C. [106]; E.C [107,118,124,126,127,130]). All 27 cases contained lesions centered within the parietal lobe, but it should be noted that a minimal number of cases were reported to contain lesions that extended slightly beyond the temporo-parietal junction (6/27) or the parieto-occipital sulcus (5/27).

Lesion hemisphere was not specified in the inclusion criteria, nor the search terms used across databases. Yet, all 27 cases described here are LH lesions. We found only two RH cases that met our criteria, but both cases exhibited ambiguity due to participant handedness (one subject was stated to be left-handed and likely cross-lateralized [39] and the other was ambidextrous following forced right-handed writing) [28]. This was an unexpected finding based on the prevalence of bilateral and right hemisphere parietal lobe interest in numeracy literature and is addressed in the discussion (4.3). Because of the limited quantity of RH cases and the lack of certainty surrounding the functional role of the hemisphere in both cases, we decided to focus this review on the LH exclusively. Thus, all analyses going forward were conducted with a LH region-of-interest and results were interpreted with this perspective.

### 2.2. Functional IPS mask

**Fig. 2.**
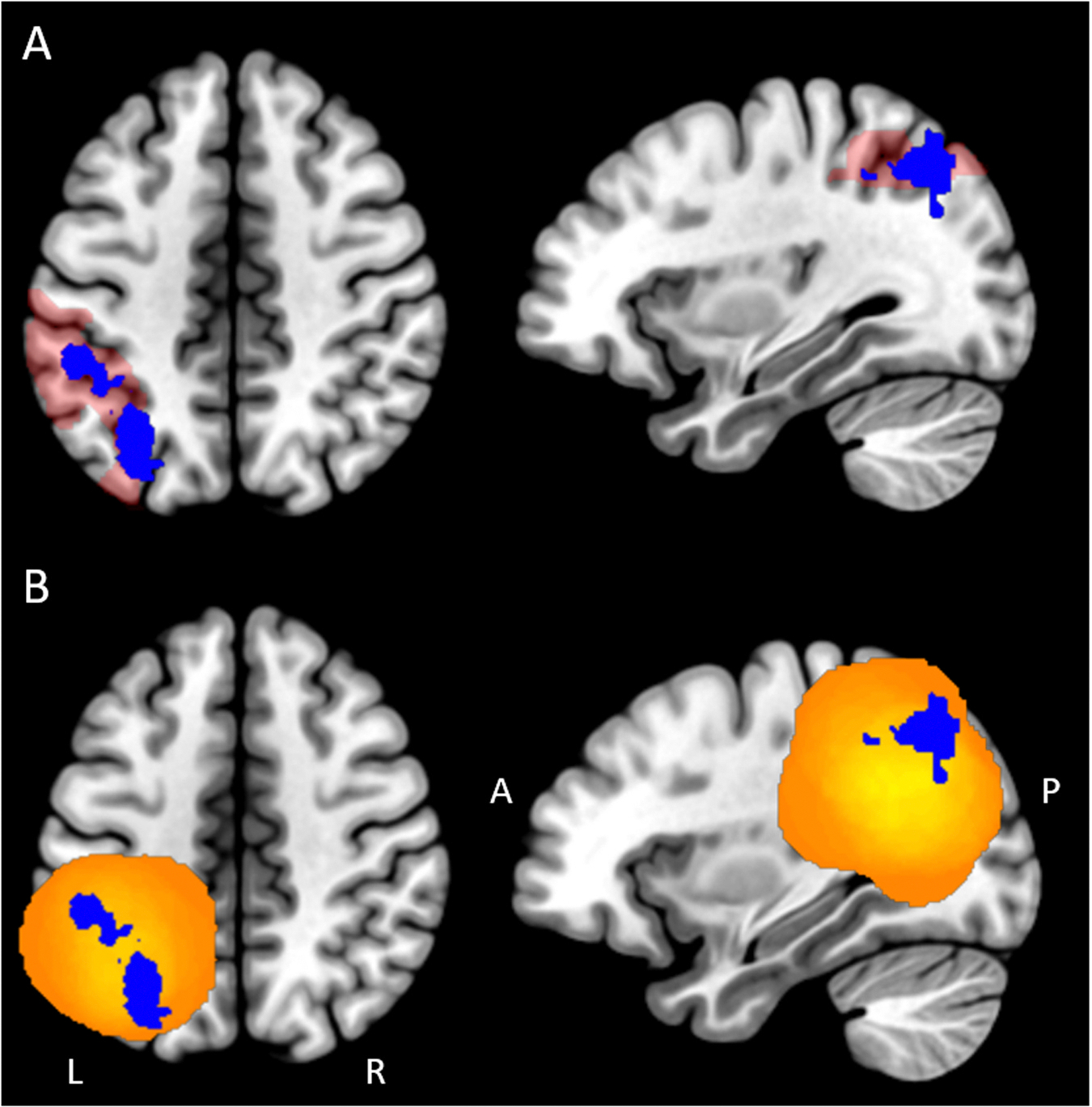
Total lesion distribution of case spheres and left intraparietal sulcus mask. (A) Functional left intraparietal sulcus (IPS) mask derived from a combination of Neurosynth Topic 18 and terms “arithmetic” and “calculation” in blue (B) IPS mask overlaid with total lesion distribution of spheres drawn for all 27 cases; brighter yellow indicates voxels encompassed by a greater number of spheres.

To assess the left IPS, we used meta-analytic information within the Neurosynth database [132] to derive an IPS mask in the left hemisphere. Our goal was to create a mask located along the anatomical contours of the IPS with strong evidence for a functional role in numeracy. The functional clusters were extracted from: 1) Neurosynth v5-topics-100 meta-analysis Topic 18, which includes studies that contain terms like “magnitude”, “estimation”, and “number”, and 2) key term searches for a) “arithmetic” and b) “calculation.” This allowed us to closely assign our IPS mask to prominent theories, as it was derived from a breadth of functional numeracy studies rather than only one term. The IPS cluster was created by identifying the largest LH parietal cluster in the three Neurosynth statistical activation maps, then selecting only voxels that overlapped in all three. The resulting mask was compared against an anatomical atlas correlate (Automated Anatomical Labeling atlas; AAL) to confirm that it centered around the contours of the anatomical sulcus. Visualized in Fig. 2A, the final cluster was used as the functional IPS mask in all analyses.

### 2.3. Assignment of cases based on evidence of IPS damage

To address our primary hypotheses about the specific role of IPS in numeracy abilities, we assigned each identified case to one of two groups: cases with strong evidence of IPS involvement (IPS group), and cases without (Other Parietal group). More specifically, we used a conservative, dual-criteria approach to determine assignment of cases to the IPS group. The first criterion was based on the original written description of the lesion location: it involved reviewing the written description for each case to determine whether it specifically noted the IPS as part of damaged brain tissue. The second criterion was based on the provided neuroanatomical image(s) of the lesion location: as described below, it involved a template-based approach in the Montreal Neurological Institute (MNI) atlas space to determine whether the visual evidence of the lesion site supported a probable localization inclusive of numeracy-related IPS tissue.

To implement our template-based approach, we first carefully examined the provided images of each case to visually infer its center-of-mass and spatial extent. Then, using sulci and gyri as markers, we selected the best-corresponding location within the standard MNI152 brain template and defined a spherical region-of-interest (ROI) centered at this corresponding location. The sphere diameters were adjusted to encompass the estimated spatial extent of the lesion. This approach was selected in favor of tracing a single slice and extrapolating to a 3D space because it allowed for a standardized protocol for estimating the spread of each lesion when individual cases were presented on different planes and varied in total lesion volume, as well as differed in the number of images provided to produce an accurate and complete representation of the lesion. As a final step, we determined whether the spherical ROI representing the lesion within the MNI brain template encompassed numeracy-related IPS tissue. For each case, we computed the overlap between our functional left IPS mask and the spherical ROI. To be considered for the IPS group, cases had to have at least one overlapping voxel.

Details about the implementation of our criteria for group assignment are provided in Table S1, which summarizes extracted information based on the original written description of the lesion, and Fig. 2B, which demonstrates the total spread of all 27 cases relative to our IPS mask. The resulting IPS group (*n* = 8) includes cases that:1) specifically indicated that the lesion included the IPS in the original written description and image, and 2) had overlap of the created ROI sphere of the lesion with the IPS mask. The Other Parietal group contained cases that met neither (n = 5) or only one of our criteria (n = 14). It is important to emphasize that our classification criteria follow a more conservative approach in order to prioritize rigorous assessment of the IPS anatomically. This approach gives us high confidence that all of the cases in the IPS group definitively involve lesions to the IPS, though it also allows for the potential existence of a degree of ROI overlap in the Other Parietal group. For more detail about the spheres generated for each case, see Fig. S1. The 3D spherical ROI files for each individual case will be uploaded to an OpenNeuro repository upon publication.

### 2.4. Behavioral performance

To examine behavioral impairments, we compiled numeracy task performance into four main categories: Approximation, Calculation, Ordinality/Cardinality, and Transcoding. These categories are frequently assessed in numeracy literature, probe theoretically distinct numeracy domains, and may crucially involve different regions of parietal cortex [77,84,95,98]. Additionally, the four categories and included tasks are in line with four numeracy task functions previously proposed for diagnosis of acalculia [24]. The tasks described in each article were categorized as follows: Approximation assessed the ability to approximate magnitude and included dot estimation or Arabic numeral (symbolic) and dot (non-symbolic) comparison tasks, specifically requiring the participant to identify a difference in magnitude and select the larger or smaller value without requiring exact quantities. Approximation cases were grouped together based on prior studies that have found comparable activation of the left parietal cortex in both symbolic and non-symbolic tasks [21,48,51,83,96,128]. Though, we acknowledge there is some theoretical debate in magnitude estimation literature that dispute the processing differences between symbolic and non-symbolic numerical stimuli [60,117,133], so we statistically supported our decision with an analysis comparing item presentation (symbolic versus non-symbolic) (see 2.5). Calculation assessed precise operations, encompassing addition, subtraction, multiplication, and division problems in simple (i.e., single digit) and complex (i.e., multidigit, fractions, or decimals) forms. Ordinality/Cardinality assessed participants’ ability to count and understand number sequences. This category included precise enumeration of an object set, counting sequences, number ordering, or numerical labeling of positions on a number line. Though ordinality and cardinality are theoretically distinct processes, both assess symbolic number relationships through similar behavioral tasks and were not frequently assessed individually across cases, so we have chosen to combine them for analysis. Transcoding assessed the ability to convert a number from one form to another, including tasks with oral to written forms, or Arabic numeral to written word inputs and outputs.

All numerical measures performed in each case study were coded into one of the four numerical categories (or Other, and not included in this analysis), and scores were binarized for each measure. Binarized scores were determined through the original article’s reported scores and/or definition of impairment for that individual. For example, if a score was described as “relatively intact” for a case, that category was inferred to be intact (binarized score = 0); in contrast, scores that were highlighted by the author as below average, impaired, or abnormal were considered to be impaired (binarized score = 1). Then, the average binarized score was calculated for Approximation, Calculation, Ordinality/Cardinality, and Transcoding for each case subject. Cases that received an average binarized score > 0 were considered Impaired in that category. The complete coding of numerical measures, original scores and descriptions, and binarized scores is available in Supplemental Materials as a.txt file.

For a complete view of each case’s profile, we additionally noted the absence or presence of aphasia and Gerstmann’s Syndrome for each case because aphasia is often comorbid with acalculia, and Gerstmann’s Syndrome includes acalculia as one of four diagnostic criteria [4,56]. Each comorbidity was determined either directly by the authors, or through clear listing of symptoms (for aphasia, noted difficulty with word finding, speech, and/or reading; for Gerstmann’s Syndrome, the combined presence of acalculia, agraphia, left-right confusion, and finger agnosia).

During behavioral data collection, for cases in which participants were assessed at multiple timepoints with repeated administration of assessments, data points were taken from the most chronic timepoint. Chronicity of lesions was noted and initially assessed for group differences. A chronic lesion was considered to be 5 months or greater since the time of stroke, with cases less than 5 months considered in the acute phase. A summary of demographics across the full sample and for IPS and Other Parietal damage groups is shown in Table 1.

### 2.5. Primary statistical analyses

To determine whether chronicity of lesions would impact our behavioral analyses, cases were labeled as either Acute or Chronic lesion cases, based on the latest available timepoint post-stroke for each case. We calculated a total deficit ratio for each case (sum of impairment across domains / total tested domains) and performed a two-tailed *t*-test with total deficit ratio as the dependent variable to assess mean frequency of behavioral deficits between the two chronicity groups. This test showed no significant difference between acute and chronic lesion cases, so chronicity was not included as a covariate in domain-specific analyses.

In order to compare numeracy performance between our IPS and Other Parietal groups, we performed a Fisher’s Exact Test comparing the frequency of impaired versus intact performance across these two groups, separately for each task category. Fisher’s Exact Test, instead of the more common and conceptually similar Chi Square test, was selected because it is recommended when small sample sizes result in an expected value of less than 5 for one or more conditions of the contingency table [74]. For each category (Approximation, Calculation, Ordinality/Cardinality, Transcoding), a 2×2 contingency table was created with lesion group as one factor (IPS or Other Parietal) and the other factor as behavioral performance in that task category (Intact or Impaired). Note, to statistically support our decision to combine symbolic and non-symbolic approximation tasks as one domain, we compared Approximation performance for symbolic magnitude comparison and non-symbolic magnitude comparison individually as well. All p-values were corrected for multiple comparisons using the Bonferroni method (uncorrected α =.05; corrected α =.0125). Statistical analyses were completed and visualized using R 4.2.0 software [101].

### 2.6. Exploratory analysis

As a follow-up analysis to examine possible anatomical associations with distinct domains of numeracy, we performed activation likelihood estimation (ALE) contrast analyses for each task category, using the software BrainMap GingerALE 3.0.2 [46,47,121]. ALE was developed to support meta-analyses of coordinate loci corresponding to significant activation clusters in functional neuroimaging studies, by using a Gaussian distribution to create a probabilistic voxelwise map of likely activation based on given coordinates [121]. The original use involved compiling standard coordinates describing peaks of activation from a set of published studies, and then modeling and summing the probabilistic distribution of activation spreading from the central point of each reported coordinate locus. Here, we similarly used the center coordinates of the spherical ROIs representing each lesion in the MNI brain template as a set of “lesion loci” and then employed the ALE analysis to model and sum the probabilistic distribution of lesion loci correlated with behavioral impairments. This allowed us to quantify the probability of anatomical differences in lesion location (as an inferred center-of-mass) between cases that exhibited intact versus impaired behavioral performance for each of our task categories. This approach is a novel and exploratory use of ALE, but it benefits from a probabilistic distribution accounting for the degree of uncertainty that results from estimating lesion locations based on the limited neuroanatomical information provided in single case reports. Importantly, it provides a quantitative method for aggregating information about lesion location across cases.

To implement our ALE analyses, coordinates were defined as the center-of-mass of each spherical lesion ROI within MNI space, and all analyses were run using the MNI152 brain template. Single ALE analyses were run for Intact or Impaired behavioral performance with a p-value of .05 and 0 mm^2^ minimum volume clusters for each group to create thresholded ALE maps. The two thresholded group ALE maps for each task category were then compared in a contrast ALE (P-value =.05; 0 mm^2^ min. vol; 1000 permutations). The same procedure was employed for aphasia and Gerstmann’s Syndrome as an exploratory investigation of how these comorbidities may align with task categories. All maps were visualized using MRIcroGL software (v1.2.20220720) [105].

## 3. Results

### 3.1. Behavioral patterns between IPS and other parietal

Our primary goal was to compare numeracy performance across single case studies with left IPS or other left parietal damage through the lens of the prominent Triple Code Model and Approximate Number System theories. For each case, we coded numeracy performance separately for four task categories corresponding to theoretically distinct aspects of numeracy: Approximation, Calculation, Ordinality/Cardinality, and Transcoding. Table 2 provides a full profile of the individual demographics and coding of behavioral performance of all cases for each task category. We did not find a significant difference in frequency of total behavioral impairment between acute and chronic lesion cases (*t* (21.59) = −0.64, *p* = 0.53), so chronicity was not included as a covariate in further analyses and all cases were considered together.

**Fig. 3.**
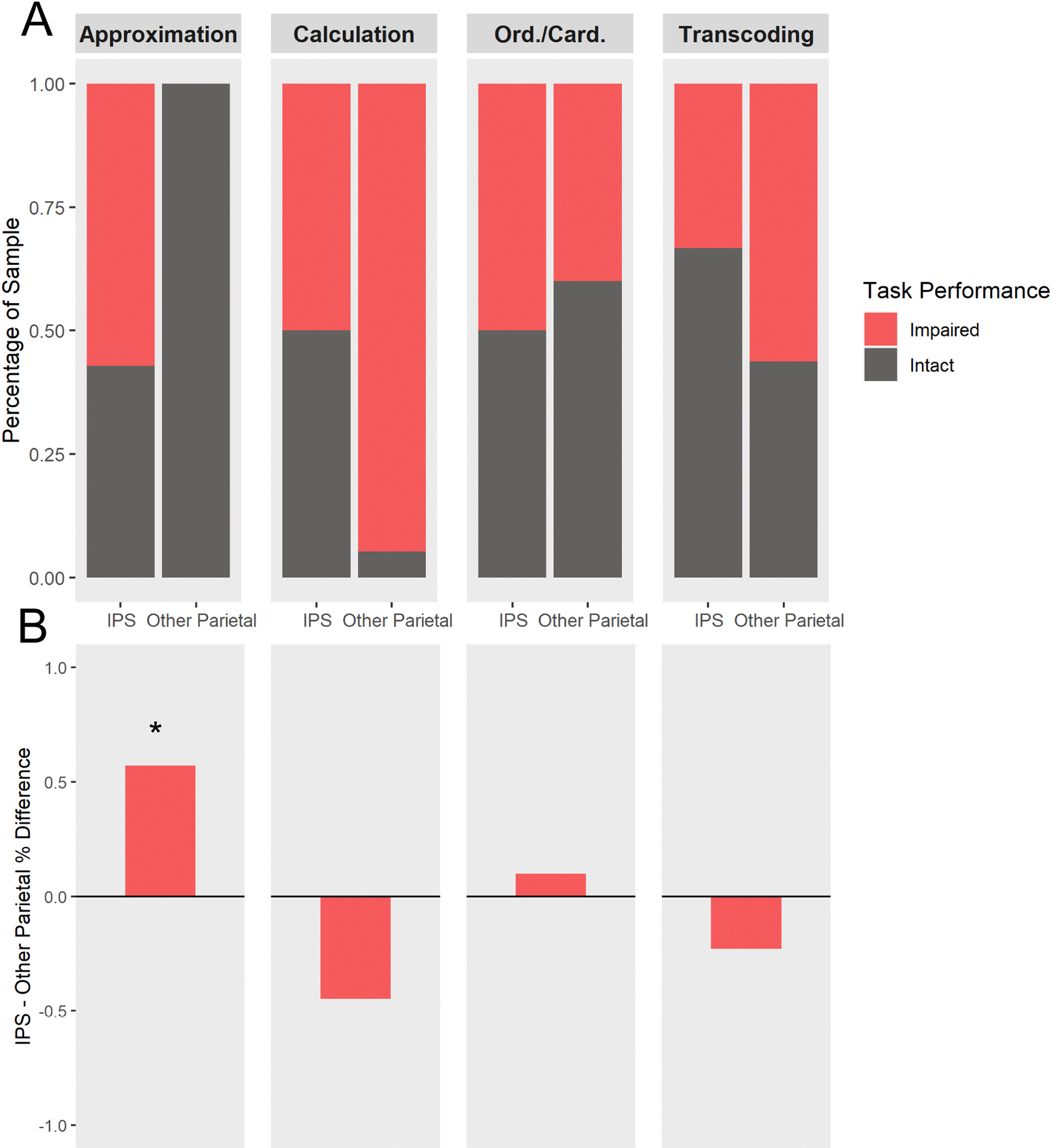
Visualization of Fisher’s Exact Test grouping for each numeracy task category. (A) Impaired (red) versus Intact (gray) performance shown as a percentage of the total sample in each task category (Approximation n = 25, Calculation n = 27, Ordinality/Cardinality n = 19, Transcoding n = 22). (B) The difference between IPS Impaired percent of sample minus Other Parietal Impaired percent of sample for each task category. Significant result indicated with asterisk (Bonferroni corrected α <.0125).

To assess the frequency of performance impairments in each task category between the IPS and Other Parietal cases, Fisher’s Exact Tests were carried out for each category, and corrected for multiple comparisons (Fig. 3). Most interestingly, Approximation impairments were uncommon across the sample size (4 of 25 tested cases), and all of these cases were in the IPS group, a case distribution highly unlikely to occur by chance (*p* = .003). These results suggest that damage to the left IPS can be sufficient to produce impaired performance on tasks involving approximation and estimation skills. When the Approximation category was split by item presentation (symbolic versus non-symbolic) to assess theory-based task differences, both symbolic Approximation impairments (*p* = .005) and non-symbolic Approximation impairments (*p* = .012) were significantly more frequent in the IPS group.

Calculation tasks were the only category clearly assessed in all 27 case studies and impairments were frequently observed (22 out of 27 cases). The Calculation result was above the corrected threshold, but notably the trend indicated a higher frequency of calculation impairments in the Other Parietal group, relative to the IPS group (*p* = .017). We found that no significant difference emerged for Ordinality/Cardinality (*p* = 1.0) or Transcoding (*p* = .64). Ordinality/Cardinality impairments occurred in 8 of 19 test cases and Transcoding impairments occurred in 11 of 22 tested cases. Notably, Ordinality/Cardinality was the least tested task category, possibly due to the challenge of distinguishing the task features from other elements of numeracy. Taken together, the results of our primary analysis emphasize the involvement of the left IPS in approximation ability, but do not support a relationship between damage to the left IPS and impairment in calculation, ordinality/cardinality, or transcoding.

### 3.2. ALE contrasts

**Fig. 4.**
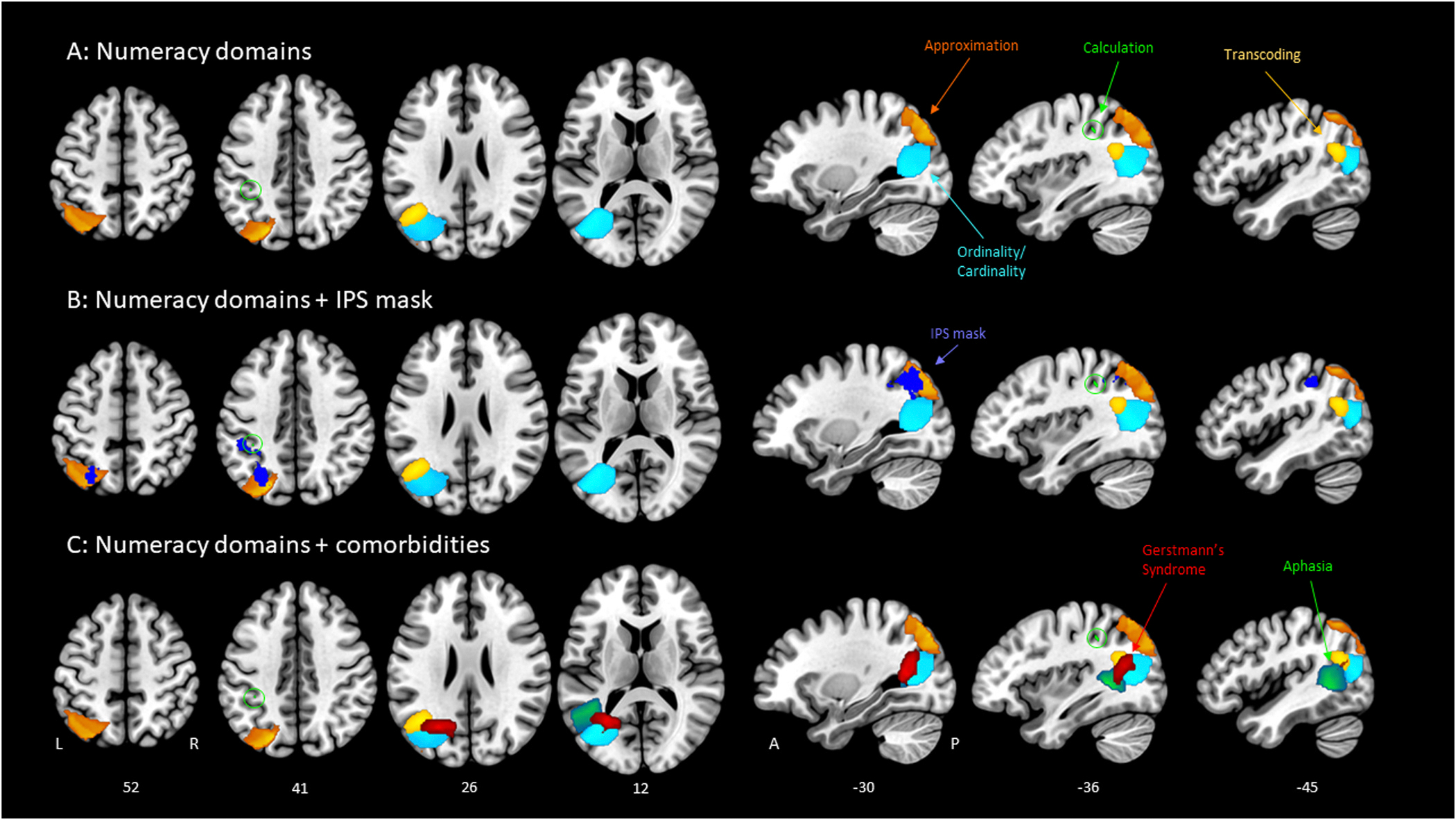
ALE contrasts for deficits in numeracy domains and co-morbidities relative to the left intraparietal sulcus. (A) Results of ALE contrasts for deficits in numeracy domains (Approximation, Calculation, Ordinality/Cardinality, Transcoding). Each cluster survives a p < .05 threshold and contains a range of Z scores corresponding to the brightness of cluster color (Z-score ranges vary individually but range overall from Z = 1–2.75) (B) Four numeracy domains + the functional IPS mask derived from Neurosynth (C) Results of ALE contrasts for comorbidities of aphasia (Z = 1–2.75) and Gerstmann’s Syndrome (Z = 1–2.37) overlaid on the four numeracy domains.

Our primary analysis provides evidence for the left IPS’s role in approximation performance specifically, but indicates that the other three numeracy domains may instead crucially involve other regions of the left parietal cortex. As a follow-up exploratory analysis, we explored the neuroanatomical correlates of our behavioral task categories beyond the left IPS. We employed a contrast ALE analysis approach for each task category to determine areas of significant probabilistic overlap for impaired performance in the respective domain. It should be noted that this approach does not allow for factors like lesion volume to influence results and thus may not capture total spread of some lesions, but does weight the centers of mass equally. We observed a significant cluster (*p* < .05) for each contrast (Impaired – Intact), with neuroanatomical differences in their location (Fig. 4).

The results of this exploratory analysis converge with our primary analysis. Specifically, we observed an Approximation Impaired cluster (Z = 1.00–2.75) as the only cluster to overlap with our functional IPS mask (Fig. 4B). Anatomically, this Approximation cluster encompasses portions of the left superior parietal lobe and posterior inferior parietal lobe, including the posterior IPS that also fall within the functional IPS mask. Peaks of the cluster are localized to the central and posterior portions of the left inferior parietal cortex on a more lateral plane, but converge on posterior IPS when moving medially through the brain.

The results for the Calculation Impaired contrast shed further insight on the results obtained from our primary analysis. Specifically, the Calculation Impaired cluster (Z = 1.00–1.67) is the weakest of our observed clusters, even though statistical power for this contrast should be highest since calculation performance was assessed in all cases. The locus of the cluster falls in the anterior portion of the left inferior parietal cortex, close but not overlapping with our IPS mask in this analysis, which lends some support to the idea that the left IPS may support estimations of quantity that can assist with precise calculation [20,70]. Of note is the fact that Calculation was the only category assessed in all 27 cases, and the resulting weak cluster from a contrast (Impaired – Intact) implies that there may be a strong degree of overlap between lesion location of cases with intact and impaired calculation performance. Overall, these results may indicate a more complex network for calculation processing, as behavioral performance does not appear to be confidently linked to one particular brain region in this analysis. Such a result would be in line with prior studies linking a greater variety of regions to calculation abilities [9,65] and the notion of a ‘secondary’ acalculia that can result from non-numeric cognitive impairment [7,19].

Interestingly, the Ordinality/Cardinality Impaired cluster (Z = 1.00–2.37) shows the greatest degree of cohesion based on consistently high Z-scores per voxel, indicating a focused alignment with the white matter underlying the left temporo-parieto-occipital junction and middle occipital gyrus, extending laterally to the posterior portion of the middle temporal gyrus. When comparing the numeracy domains to the possible co-morbidities of Aphasia and Gerstmann’s Syndrome (Fig. 4C), there is overlap between Ordinality/Cardinality and both comorbidities, but particularly with Gerstmann’s Syndrome. The Gerstmann’s Syndrome cluster is localized to the left middle occipital gyrus and the dorsomedial parietooccipital sulcus, with the most superior portion overlapping with the angular gyrus. This finding is in line with prior research on the angular gyrus and, in particular, its association with Gerstmann’s Syndrome [6,110,122]. Our results suggest that there may be a potential connection between Gerstmann’s Syndrome and Ordinality/Cardinality impairment.

Finally, the Transcoding Impaired cluster (Z = 1.00–2.46) overlaps with the left angular gyrus, posterior inferior parietal lobe_,_ and a portion of the posterior region of middle temporal gyrus. This domain resulted in a relatively small significant cluster, which may be due to fewer distinct voxels correlated with impairment over intact performance on Transcoding tasks. The comorbid Aphasia cluster, localized to the left posterior middle temporal gyrus and temporoparietal junction, was in a similar area of the brain. Given that Transcoding tasks involve the greatest degree of verbal processing through oral and written inputs and outputs, this finding is anatomically logical.

## 4. Discussion

Our aim was to compile numeracy-focused single case studies for direct comparison and application to prevailing theories of numerical processing. According to the Triple Code Model, the inferior parietal cortex and, particularly, the bilateral IPS are crucially involved in magnitude processing. Magnitude processing, through the approximation of quantity, is thought to undergird learned numeracy skills involving symbolic numbers and learned procedures as described in the Approximate Number System model. Thus, we focused on single case studies with parietal lesions to assess the role of the IPS across a broad range of key numeracy domains. We expected to assess parietal lesion cases bilaterally, based on prior case series that implicate the left and right hemispheres in distinct yet related phenotypes of acalculia. Surprisingly, we identified 27 LH cases that met inclusion criteria, but only 2 RH cases, and therefore proceeded to conduct all analyses with a left hemisphere focus exclusively. For our primary analyses, we used Fisher’s Exact Tests to assess the significance of behavioral impairment frequency between cases with and without clear evidence of left IPS damage. Our findings support the Triple Code Model because we found evidence that damage to this region is crucial to produce impairments on tasks involving approximation, but we did not find evidence specifically associating left IPS damage with impaired performance on other types of numeracy tasks. These primary findings led us to conduct exploratory ALE analyses, which provided convergent evidence that left IPS selectively supports approximation tasks, and additionally yielded suggestive evidence of left angular gyrus and temporo-parieto-occipital junction involvement in the performance of transcoding and ordinality/cardinality tasks, respectively. Weak statistical results were observed for all analyses involving measures of calculation, aligning with evidence that calculation processing employs a distributed set of regions.

### 4.1. The role of the IPS distinct from parietal cortex

The foundational role of the IPS outlined in the Triple Code Model and Approximate Number System theories centers around magnitude processing and innate approximation ability. The results of our Fisher’s Exact tests are in line with this theory. We found that Approximation performance was more likely to be impaired in left parietal cases categorized into our IPS Group versus Other Parietal. This aligns with both the foundational argument of the Approximate Number System and the Triple Code Model’s view of an *analog magnitude representation* code centered around the IPS in the inferior parietal lobe [35,38,39]. These results were further supported by the exploratory ALE contrast results for the Approximation impairment cluster, which demonstrated overlap with the posterior IPS. In contrast, Fisher’s Exact results for Transcoding and Ordinality/Cardinality were not strongly associated with damage to either group, while Calculation impairment was trending toward a greater frequency in the Other Parietal group without definitive IPS damage. Interestingly, such an organization echoes the theory of three parietal circuits that Dehaene later proposed [41], in which distinct parietal regions are underlying components of quantity processing beyond the original Triple Code Model.

Our results support the importance of left IPS in approximation ability, but this is not without a degree of complexity. One of the interesting behavioral patterns to emerge across cases was a split in behavioral phenotype in the IPS group. Four of eight IPS cases were impaired in Approximation performance, while the other tested cases were intact according to each article’s criteria. This pattern is surprising because it suggests that the relationship between left IPS damage and magnitude processing is not clear-cut. While approximation impairment is more likely to occur following damage to the left IPS specifically (in fact, no Other Parietal cases demonstrated an Approximation impairment), it may not necessarily occur. Such a pattern implies that the left IPS is important for approximation and magnitude representation, but damage to the left IPS may not be sufficient for a total loss of function in this behavioral domain. Future studies may examine the mechanism behind this finding, perhaps exploring intact right IPS compensation.

In this study, we chose to combine symbolic and non-symbolic approximation tasks on the grounds of prior research and our statistical findings between the two stimuli forms. The results of the Fisher’s Exact Tests showed the same pattern of greater frequency of impairment in the IPS group for both symbolic and non-symbolic magnitude approximation tasks individually, and these aligned with the combined Approximation results. This is in line with prior research that finds activation of the left IPS during both forms of stimuli [21,48,51,83,96,128]; however, our results do not speak to the possibility of stimulus-specific activation patterns within regions of the IPS, or distinct neuronal populations associated with each item presentation format [2,83,90,116,120]. The exploratory findings of the ALE analysis implicate the posterior IPS, in particular, with Approximation deficits. Posterior IPS has been previously implicated in both symbolic and non-symbolic numeracy tasks as a ROI distinct from IPS as a whole region [26,59,115,128]. Because this analysis combined item presentation format, the emerging significant cluster could be considered a region involved in the underlying magnitude representation that is common between symbolic and non-symbolic comparison tasks. The two formats may also exhibit distinct activations across the left IPS as a whole, as well as hemispheric differences, that were not assessed in the current study.

Of additional interest is the pattern of calculation performance relative to the left IPS that emerged in our results. Only five cases across the dataset exhibited intact calculation abilities, and four of those cases were individuals in the IPS group. Most importantly, the IPS cases demonstrating intact calculation were the same four individuals with intact approximation. In contrast, the four IPS cases with impairment in approximation also demonstrated impairment in calculation performance. This points toward an interesting role for the left IPS and its relationship to domains of numeracy beyond approximation. Calculation performance has been linked to the IPS through prevailing numerical processing models [38], but calculation impairments have been identified following frontal lesions as well [82,119]. Prior fMRI studies have found that the network responsible for calculation processing requires a high cognitive demand for additional skills beyond basic number sense and, consequently, implicates brain regions both within and beyond the parietal lobe [9,65]. This idea can be further supported by the notion of primary versus secondary acalculia phenotypes. Essentially, primary acalculia is considered a ‘pure’ deficit of numerical processing, while secondary acalculia results from impairments in other cognitive demands, such as working memory, attention, or visuospatial processing [7,8,19]. Our results suggest that a calculation deficit can emerge when underlying approximation ability is compromised through left IPS damage, following the phenotype of primary acalculia, but other mechanisms beyond magnitude processing are also viable sources of calculation impairment when approximation performance remains intact, resulting in secondary acalculia. This is evidenced by the 18 impaired calculation cases in the Other Parietal group, who demonstrate a phenotype of impaired calculation with intact approximation. The concept of secondary acalculia could be further expanded through a larger sample within each behavioral domain to statistically examine the observed pattern, as well as an exploration of lesions beyond the left IPS to explore hemispheric differences and the impact of damage to non-numeric cognitive mechanisms on calculation performance.

### 4.2. Exploring the parietal lobe

In order to examine the neuroanatomical correlates of Calculation, Transcoding, and Ordinality/Cardinality, which were not found to be significantly associated with IPS-specific damage, we employed an exploratory contrast ALE analysis for all categories and the possible co-morbidities of aphasia and Gerstmann’s Syndrome. It is important to note that while these findings are exploratory and should be considered as such, the use of ALE analysis to investigate structural datasets has been employed previously outside of the lesion method (for a review, see [53]). Here, we use ALE to novelly aggregate across lesion cases that would otherwise lack a quantitative method for investigation. We acknowledge that our analysis is limited by a small number of foci input into the ALE analysis, as well as the center-of-mass inference required to standardize our case lesions using a template-based approach [53,89]. However, we employ a stepwise approach that parallels the steps required for standard ALE meta-analyses with functional datasets, and we are confident that our exploratory results support and strengthen the findings of our primary analysis.

Through our experimental use of ALE with lesion center-of-mass coordinates, we found that distinct clusters emerged for the Impaired group of each domain. Notably, Approximation was the only cluster to overlap with our left IPS mask. Transcoding was localized to the left angular gyrus, inferior parietal lobe, and a small posterior portion of the middle temporal gyrus. Ordinality/Cardinality was localized to the left temporo-parieto-occipital junction and middle occipital gyrus, with some spread anteriorly to the posterior portion of the middle temporal gyrus and superiorly toward inferior parietal cortex. Calculation was localized to the left anterior inferior parietal cortex, though the cluster was the weakest of the domains and is perhaps a better representation of the wide-spread nature of calculation processing, given little difference in lesion location between Impaired and Intact groups. When compared to the significant clusters of aphasia and Gerstmann’s Syndrome, Transcoding impairments expectedly overlapped with Aphasia as the task category requiring the greatest verbal demands, while the category most closely overlapping with Gerstmann’s Syndrome was the Ordinality/Cardinality cluster. The latter association is less expected, though given the anatomical location and the visuospatial nature of the mental number line utilized in ordinality, it is reasonable that ordinality/cardinality processing would be linked to a disorder involving several spatial impairments (e.g., left-right confusion) [14,56].

Though our anatomical findings are exploratory in nature, the identification of distinct clusters for specific categories of numerical processing lends support to prior theories. With respect to the Triple Code Model, our anatomical findings demonstrate a dissociation of different components of numerical processing in a pattern not dissimilar to Dehaene’s proposed behavioral areas [38,39]. The left inferior parietal lobe was implicated in Approximation, aligning with the *analog magnitude representation* component. The notion of a *verbal auditory word frame* in the perisylvian area most closely aligns to the location of the Transcoding cluster in the posterior portion of the left middle temporal gyrus and angular gyrus. Ordinality/Cardinality, which involves visual representations of numbers in the number line and localized to the left temporo-parieto-occipital junction, is perhaps the closest match to the *visual Arabic number form* representing visual Arabic numerals, but this code aligns least clearly with our results.

Interestingly, while our findings generally align with the original Triple Code Model, the neuroanatomical locations from the ALE analysis correspond even more closely with Dehaene’s alternate model of three parietal circuits [41]. Our findings strongly align with Approximation localized to the IPS for quantity processing and Transcoding localized to the angular gyrus for verbal number processing. The region proposed to underlie the spatial element of the mental number line is suggested to be in posterior superior parietal cortex, though our behaviorally comparable Ordinality/Cardinality cluster is located more inferiorly near the temporo-parieto-occipital junction. In Dehaene et al. [41], this element is the most speculative component of the model, and is suggested to be involved in tasks including approximation as well, which does align with the superior posterior cluster of our Approximation results.

### 4.3. Hemispheric differences in case study acquisition

The current review is limited to a left hemisphere focus due to the available single case studies that fit our inclusion criteria. We did not originally select LH cases during the literature search. We ultimately decided to do so because only two RH lesion cases fit our criteria, and both had ambiguous lateralization due to patient handedness. We believe that there are two possible explanations, that are not mutually exclusive, to account for the pattern.

First, there may be true hemispheric differences in the likelihood of acquiring acalculia following brain damage. The identification of significantly fewer RH cases in our literature search may imply that there are simply fewer RH cases exhibiting notable impairments across major numeracy tasks. The Triple Code Model, in its neuroanatomical specifications of components of numerical processing, attributes either bilateral or left-lateralized locations to its codes [38]. Theoretically, this implies a greater involvement of the left hemisphere, relative to the right, in processes that result in acalculia when damaged. Across case series, LH lesion groups consistently demonstrated greater impairments than RH cases across a range of calculation, transcoding, and counting tasks [44,63,79,109], aligning with a “primary” phenotype of acalculia [19]. Thus, it may be the case that damage to the left hemisphere more frequently results in impaired numerical performance.

Second, the left hemisphere skew of single case studies in the literature may result from the patient acquisition stream. Cases are frequently found through clinical assessment following brain injury in aphasia-related clinics. Therefore, individuals without aphasia have an increased likelihood of flying under the radar. Given that left hemisphere strokes more often result in aphasia due to the left lateralization of language [18], it is possible that this allows for more numeracy-impaired individuals to be identified than in right hemisphere, non-aphasic cases. Clinical aphasia assessments typically include limited numerical focus, focusing exclusively on symbolic number representation. An example of this is the base Western Aphasia Battery-Revised (WAB-R) without supplementary tasks, which includes a six-item auditory word recognition subtask of transcoding spoken number words to the Arabic numerals on the page, three instances of verbal repetition of numbers, and one real world application asking for the number of days in a week. Prior research more strongly implicates the left hemisphere with verbal, symbolic numeracy tasks like those found in the WAB-R [29,109,113,114,129], while, in contrast, the right hemisphere is more often associated with a non-symbolic magnitude role, typically centered around visuospatial and procedural ability in numerical processing [12,29,49,64,114]. As a result, acalculia resulting from right hemisphere lesions may not be effectively tapped with the current symbolic, aphasia-focused measures. For individuals with impaired numerical processing through spatial effects as a result of a right hemisphere lesion, many current assessments lack the ability to detect such problems at all. In fact, stroke survivors themselves have reported a lack of knowledge about their own deficits post-stroke, because of a lack of awareness of acalculia as a possible condition [13]. Therefore, right hemisphere cases may largely be overlooked in the case acquisition pipeline, resulting in a reduction of representative cases published. Ultimately, the numerical processing picture is not complete without a solid understanding of acalculia following both left and right hemisphere damage. Future studies must consider hemispheric differences and the sensitivity of acalculia assessment measures moving forward, as the role of each hemisphere and their interactions with one another following damage will shed light on the fundamentals of number processing in the brain.

### 4.4. Considerations regarding the use of case studies

It is important to consider the benefits and limitations of case studies when assessing the literature. Case studies often result from identification of a notable phenotype, and considering multiple cases together can allow for impactful investigation of the prevalence of such phenotypes. Further, aggregating cases can be effective for revealing unexpected patterns, providing evidence of generalizability across individuals with differing demographic backgrounds, and generalizing across conceptually related assessment measures. Finally, accumulation of case studies can provide a greater sample size, and a richer set of assessment measures, for assessing a particular region as compared to studies that use voxel-based lesion-symptom (VLSM) methods to evaluate structure-function correspondences. VLSM studies acquire stroke participants without discriminating lesion location, and are thus susceptible to under sampling areas that are less frequently impacted by stroke (such as the parietal lobe).

However, the benefits that come from aggregating single case studies must be counterbalanced by the limitations that come with the single-case method. One limitation is a sampling bias. We believe that patients with impairments in calculation are much more likely to be identified and explored as an acalculia case, as compared to patients with impairments limited to other domains of number ability. This is because bedside testing and more formal neuropsychological assessments of numeracy have historically relied upon measures limited to number naming and writing, and arithmetic calculation (such as the math subtests of the Western Aphasia Battery, with impairments in number naming and writing studied in the context of other non-numeric transcoding skills (e.g., reading and spelling) [73]. As a consequence, measures of arithmetic calculation often become the “gateway” to an acalculia diagnosis and single-case follow-up with an expanded battery of numerical tasks. This approach may neglect individuals with approximation-based deficits that do not have obvious difficulty with symbolic tasks [13]. We believe that this limitation most likely accounts for the scarcity of right hemisphere parietal cases that we identified through our systematic literature review, given the reviewed evidence supporting more frequent calculation and symbolic number format impairment in left hemisphere cases.

Similarly, we noted during the literature search that single case reports of lesions outside of the parietal lobe were relatively infrequent, despite one of our database searches allowing acalculia cases independent of lesion location. The non-parietal cases that were observed included lesions of the frontal lobe, temporal lobe, or basal ganglia. It is possible that the greater frequency of parietal lesion cases is due to influence from existing literature that motivates researchers to prioritize a parietal lesion case they may find. Because the IPS is a major focus of theoretical models of numeracy, it is possible that a sampling bias toward highlighting unusual or null results in specifically IPS-lesioned cases may arise. On the other hand, prior case series research finds a greater impairment of calculation performance in posterior lesion cases relative to anterior cases [63,109]. Therefore, the parietal bias may represent a true skew in frequency of numeracy deficits (or calculation deficits specifically) following brain damage.

Within the scope of the current study, we included both acute and chronic post-stroke cases in our analyses. This was statistically determined to not significantly impact our behavioral results, but does highlight a debate in lesion literature that offers differing opinions about the quality of behavioral assessment at different timepoints following brain damage [33]. Typically, acute behavioral data is considered to be the “purest” expression of a deficit because neural compensation has not yet had the opportunity to occur, but the full impact of brain damage is often not clear anatomically until the chronic phase. In the dataset used here, most cases provided specific information about the time of data collection relative to lesion onset, but two cases were ambiguous about the point of testing, so a confident assessment of chronicity could not be made for these instances. Ultimately, the aggregation of case studies necessitates a degree of heterogeneity, due to the different procedures, tasks, and timepoints employed by individual research groups. Despite this, we are encouraged by the robust results reported here, as they converge across our analyses and with prior theoretical findings.

### 4.5. Conclusions

Based on the pattern shown across 27 single case studies, we conclude that the left IPS is supportive of approximation ability, but the scope of its involvement in broader aspects of numeracy may be more limited than proposed by the Triple Code Model and Approximate Number System theories. Importantly, our findings suggest that approximation impairment is significantly associated with damage to the left IPS, specifically, but impairment of calculation, transcoding, and ordinality/cardinality are not. Instead, they may be more strongly localized to other areas of the parietal lobe. Thus, these findings do support a core claim of the Triple Code Model: that IPS is crucially involved in *analog magnitude representation.* However, they do not provide evidence of this magnitude representation underlying other components of numeracy performance. In fact, our findings more closely resemble the proposed revision of parietal circuitry [41], suggesting a need for further investigation of numerical processing circuits involving parietal regions. Of further note is the scarcity of right hemisphere case studies identified during the initial search, resulting in analyses focused exclusively on left hemisphere. We speculate this reflects a historical reliance on symbolic measures of calculation ability to detect acalculia, which results in the under-detection of individuals who suffer from other forms of numeracy impairments, such as impairments in approximation. Overall, the lesion method offers an important source of information to elucidate the nature of number processing in the brain. These findings should be advanced through future investigations of the right parietal case discrepancy and hemispheric differences, as well as cases bilaterally outside of parietal cortex, in order to shed further light on the neuroanatomical underpinnings of numerical processing and their relationship to acalculia following brain damage.

## Supplementary Material

1

2

## Figures and Tables

**Table 1:** Summary of demographics across total sample and for each lesion group.

	Sex	Age	Years of Education	Months since Stroke	% with Aphasia	% with Gerstmann’s Syndrome	Total Deficit Ratio

**Total Sample (N = 27)**	**M 14; F 13**	**56.89 ± 11.88**	**12.37 ± 3.65**	**17.07 ± 25.94**	**41 %**	**19 %**	**0.55 ± 0.29**

IPS group (n = 8)	M 4; F 4	55.5 ± 17.09	12.71 ± 4.15	17.43 ± 12.11	38 %	25 %	0.51 ± 0.36
Other Parietal Group (n = 19)	M 10; F 9	57.47 ± 9.44	12.17 ± 3.51	16.93 ± 29.97	42 %	16 %	0.50 ± 0.24

Mean ± SD of demographics are reported based on the information provided in each single case study. Total deficit ratio calculated as: number of numeracy categories impaired / total numeracy categories tested.

**Table 2:** Demographics, Lesion Data, and Behavioral Performance for Individual Cases.

Group	Case	Demographics					Spherical Lesions		Numeracy Categories				Co-morbidities	
		Sex	Age	Education (yr)	Time since onset	Chronicity	Center of Mass (MNI)	Sphere Size (mm)	Approximation	Calculation	Ordinality/Cardinality	Transcoding	Aphasia	Gerstmann’s Syndrome

IPS	Ashkenazi et al. [10]	M	67	15	6 m	chronic	−38, −66, 38	12	**1**	**1**	0	0	0	0
IPS	Basagni et al. [11]	M	54	~17	20 m	chronic	−30, −60, 26	26	0	0	-	**1**	0	**1**
IPS	Cappelletti et al. [25]	F	44	14	1–2 y	chronic	−28, −54, 46	22	**1**	**1**	-	0	-	-
IPS	Gliksman et al. [58]	F	22	12	1 w −2 y	chronic	−44, −38, 44	12	**1**	**1**	**1**	-	0	0
IPS	Hirayama et al. [66]	M	52	16	n/a	n/a	−36, −60, 34	20	0	0	-	**1**	**1**	-
IPS	Lemer et al. [81]	F	76	n/a	11 m	chronic	−30, −72, 24	14	**1**	**1**	-	-	0	**1**
IPS	Pia et al. [94]	F	70	5	1 m	acute	−32, −64, 22	20	0	0	**1**	0	**1**	-
IPS	Varley et al. [126]	M	59	10	3 y	chronic	−46, −36, 22	24	-	0	0	0	**1**	-

Other Parietal	Bernal et al. [15]	M	44	n/a	5 y	chronic	−42, −54, 28	20	-	**1**	**1**	**1**	0	-
Other Parietal	Chen et al. [27]	F	61	5	8 d	acute	−40, −64, 18	18	0	**1**	**1**	-	**1**	**1**
Other Parietal	Cipolotti et al. [30]	M	56	n/a	10 m	chronic	−42, −40, 38	18	0	**1**	-	**1**	0	-
Other Parietal	Cohen et al. [31]	F	55	12	2 y	chronic	−50, −38, 20	16	0	**1**	0	**1**	**1**	0
Other Parietal	Dehaene (1991)	M	41	n/a	3 y	chronic	−40, −48, 30	30	0	**1**	**1**	**1**	**1**	-
Other Parietal	Delazer & Benke [42]	F	56	8	2 m	acute	−34, −62, 26	20	0	**1**	**1**	**1**	-	**1**
Other Parietal	Hirayama et al. [67]	M	35	16	2 w – 1 m	acute	−28, −44, 44	22	0	**1**	0	0	0	0
Other Parietal	Lampl et al. [78]	M	62	n/a	5 m	chronic	−50, −38, 20	18	0	**1**	0	0	0	-
Other Parietal	Lee [80]	F	56	n/a	5 d; 42 d	acute	−48, −40, 26	18	0	**1**	-	-	0	-
Other Parietal	Marangolo et al. [85]	M	60	13	10 m	chronic	−44, −54, 20	20	0	0	0	**1**	**1**	-
Other Parietal	Polk et al. [99]	F	65	12 +	2 w; 11 m; 1 y	chronic	−50, −22, 22	10	0	**1**	**1**	**1**	**1**	0
Other Parietal	Rosca (2009a)	F	56	16	n/a	n/a	−38, −54, 30	24	0	**1**	0	**1**	0	0
Other Parietal	Rosca (2009b)	M	72	18	1 m	acute	−34, −68, 22	18	0	**1**	**1**	**1**	0	0
Other Parietal	Takayama et al. [118]	F	56	12	2 m	acute	−40, −50, 36	22	0	**1**	0	0	0	0
Other Parietal	Takayama et al. [118]	F	61	11	1 y	chronic	−32, −58, 40	14	0	**1**	0	0	0	0
Other Parietal	Takayama et al. [118]	F	62	11	2 m	acute	−28, −48, 26	12	0	**1**	0	0	0	0
Other Parietal	van Harskamp (2001)	M	74	n/a	10 y	chronic	−44, −48, 22	22	0	**1**	-	0	**1**	-
Other Parietal	Varney [127]	M	59	12	1 w; 1 m; 5 m	chronic	−32, −40, 50	14	0	**1**	0	0	**1**	**1**
Other Parietal	Warrington [130]	M	61	n/a	~1 m	acute	−40, −60, 10	20	0	**1**	-	-	**1**	-

Centers of mass in MNI coordinates (x,y,z). Performance in numeracy categories is indicated by a 1 if impairment was present in original publication, 0 if not impaired, and – if not tested. Aphasia and Gerstmann’s syndrome are included as possible co-morbidities, 1 if present according to original publication, 0 if not present, – if no information given.

## Data Availability

We have shared the behavioral data used for analysis as a Supplemental Material file (.txt).

## Appendix A. Supporting information

Supplementary data associated with this article can be found in the online version at doi:10.1016/j.bbr.2025.115453.
