# Supplementary material for: The role of the intraparietal sulcus in numeracy: A review of parietal lesion cases
Source: Behav Brain Res. Author manuscript; Available in PMC 2026 Jun 2. (PMC13229119; doi:10.1016/j.bbr.2025.115453)
Supplement: 1 [file NIHMS2170719-supplement-1.docx]

**Supplemental Table 1 Lesion Descriptions from Original Case Publications**

| **Case** | **Original Article Lesion Description** |
| --- | --- |
| Ashkenazi et al (2008) | Left IPS; minimal but not significant extension of the lesion is noted in the angular gyrus |
| Basagni et al (2021) | Left parietal lobe extending into the ipsilateral supramarginal and angular gyri; short intraparietal connections in the vicinity of the subangular region, absent in the damaged hemisphere due to lesion extent |
| Bernal et al (2003) | Left posterior temporal-parietal cortex, involving the angular gyrus, and a lacunar infarct of right thalamus. Left parieto-occipital junction |
| Cappelletti et al (2011) | Left medial, lateral and superior parietal lobe, as well as the intraparietal sulcus (IPS) were damaged |
| Chen et al (2014) | Left parietal lesion, involved the left angular gyrus and left  supramarginal gyrus, while the left intraparietal sulci (IPS) without damage |
| Cipolotti et al (1994) | Left parietal lobe |
| Cohen et al (2000) | The superior temporal gyrus, part of the middle temporal gyrus, the lower part of the post-central gyrus, the supramarginal gyrus, and the anterior half of the angular gyrus were destroyed. The superior parietal lobule was intact. Cortex buried within the IPS was partially spared. Within the IPS, the mesial cortical sheet was spared, as well as the posterior part of the lateral cortical sheet. |
| Dehaene (1991) | Left temporal/parietal/occipital hypodensity |
| Delazer & Benke (1997) | Left parietal area, including cortical and subcortical parietal regions (including area 39 and underlying  white matter, possibly including area 40) |
| Gliksman et al (2017) | Left parietal cortex in the area of the IPS with no involvement of major white matter tracts |
| Hirayama et al (2002) | Subcortical area of left postcentral gyrus, superior parietal lobe, upper portion of inferior parietal lobe, anterior edge of occipital lobe |
| Hirayama et al (2011) | Left occipital lobe, intraparietal sulcus, and the surrounding region in the inferior parietal lobule |
| Lampl et al (1994) | Hemorrhage in left parieto-temporal region; hypodense area in the left temporal lobe, including the area in the superior and midtemporal gyri |
| Lee (2000) | Hemorrhage at left parieto-temporal junction |
| Lemer et al (2003) | Hemorrhage in the left intraparietal region |
| Marangolo et al (2004) | Left temporo-parietal infarct |
| Pia et al (2009) | Middle occipital lobe, the superior occipital lobe, the PRE, the P-IPS, the P-SPL, and SLF |
| Polk et al (2001) | Subcortical white matter beneath the supramarginal and postcentral gyri of the left parietal lobe. Extended far enough inferior to potentially involve WM tracts of superior temporal gyrus |
| Rosca (2009a) | 4/3.5 cm intracranial lesion in the left parietal lobe, a mild edema and compression of the posterior horn of the left lateral ventricle. There was also a 1.5/1.2 cm left thalamic mass |
| Rosca (2009b) | Acute state showed a 3.6/3.2/3 cm left parieto-occipital hemorrhage, without mass effect |
| Takayama et al (1994) [1] | Left postcentral gyrus, the supramarginal gyrus, the anterior part of the angular gyrus, and the inferior part of the superior parietal lobule. The lesion extended into subcortical white matter |
| Takayama et al (1994) [2] | Left supramarginal gyrus and superior parietal lobule. The lesion extended into the subcortical white matter |
| Takayama et al (1994) [3] | Subcortical white matter of the left supramarginal, angular, and inferior parietal lobule |
| van Harskamp (2001) | Left middle temporal lobe and left parietal area (including supramarginal and angular gyrus, excluding most posterior/superior portion) |
| Varley et al (2005) | Damage to the supramarginal and angular gyri, with damage extending to the inferior border of the intraparietal sulcus. The superior parietal lobule was intact |
| Varney (1984) | Upper left parietal region; posterior parietal area of the left hemisphere |
| Warrington (1982) | Left posterior parietal-occipital intra-cerebral haematoma |

Distinct cases from the same publication are differentiated in brackets.

**
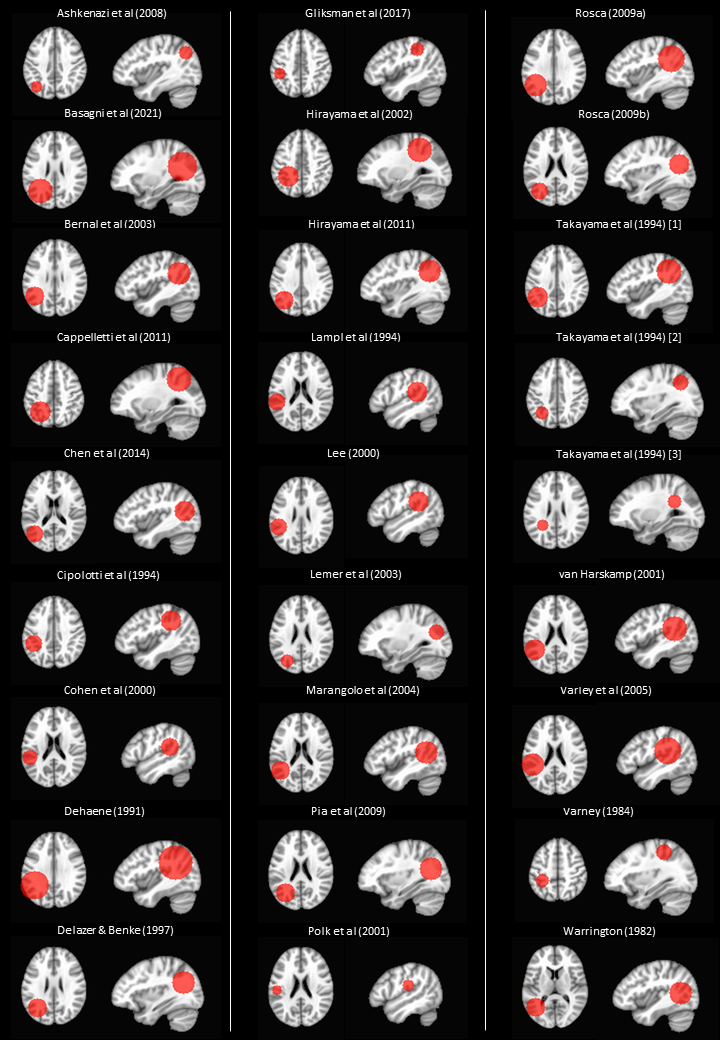
Supplementary Figure 1: Individual case spheres (*n* = 27)**. Spheres shown at center of mass on both axial and sagittal slices. Distinct cases from the same publication are differentiated in brackets.
